# Effects of non-invasive vagus nerve stimulation on attack frequency over time and expanded response rates in patients with chronic cluster headache: a post hoc analysis of the randomised, controlled PREVA study

**DOI:** 10.1186/s10194-017-0731-4

**Published:** 2017-02-14

**Authors:** Charly Gaul, Delphine Magis, Eric Liebler, Andreas Straube

**Affiliations:** 1 Department of Headache and Facial Pain, Migraine and Headache Clinic, Ölmühlweg 31, Königstein im Taunus, 61462 Germany; 2 Headache Research Unit, University Department of Neurology, Centre Hospitalier Régional de la Citadelle, Boulevard du 12ème de Ligne 1, 4000 Liège, Belgium; 3 electroCore, LLC, 150 Allen Road, Suite 201, Basking Ridge, 07920 NJ USA; 4 Department of Neurology, Ludwig-Maximilian University, Marchioninistr 15, Munich, D81377 Germany

**Keywords:** Non-invasive vagus nerve stimulation, Prophylaxis, Prophylactic treatment, Chronic cluster headache, PREVA, Attack frequency, Response rate, Patient-centric outcomes

## Abstract

**Background:**

In the PREVention and Acute treatment of chronic cluster headache (PREVA) study, attack frequency reductions from baseline were significantly more pronounced with non-invasive vagus nerve stimulation plus standard of care (nVNS + SoC) than with SoC alone. Given the intensely painful and frequent nature of chronic cluster headache attacks, additional patient-centric outcomes, including the time to and level of therapeutic response, were evaluated in a post hoc analysis of the PREVA study.

**Findings:**

After a 2-week baseline phase, 97 patients with chronic cluster headache entered a 4-week randomised phase to receive nVNS + SoC (*n* = 48) or SoC alone (*n* = 49). All 92 patients who continued into a 4-week extension phase received nVNS + SoC. Compared with SoC alone, nVNS + SoC led to a significantly lower mean weekly attack frequency by week 2 of the randomised phase; the attack frequency remained significantly lower in the nVNS + SoC group through week 3 of the extension phase (*P* < 0.02). Attack frequencies in the nVNS + SoC group were significantly lower at all study time points than they were at baseline (*P* < 0.05). Response rates were significantly greater with nVNS + SoC than with SoC alone when *response* was defined as attack frequency reductions of ≥25%, ≥50%, and ≥75% from baseline (≥25% and ≥50%, *P* < 0.001; ≥75%, *P* = 0.009). The 100% response rate was 8% with nVNS + SoC and 0% with SoC alone.

**Conclusions:**

Prophylactic nVNS led to rapid, significant, and sustained reductions in chronic cluster headache attack frequency within 2 weeks after its addition to SoC and was associated with significantly higher ≥25%, ≥50%, and ≥75% response rates than SoC alone. The rapid decrease in weekly attack frequency justifies a 4-week trial period to identify responders to nVNS, with a high degree of confidence, among patients with chronic cluster headache.

## Background

Cluster headache (CH) imposes a substantial health care burden and affects approximately 1 in 1000 individuals worldwide [1–3], with chronic cluster headache (cCH) comprising 10% to 15% of cases [4]. Patients with cCH experience many intense attacks, which have been described as the worst possible pain one can experience [4, 5]. Clinical studies and evidence-based guidelines on CH prophylaxis are limited, and few prophylactic treatment options are available for this condition [6–9]. Lithium is the only prophylactic medication currently approved for CH (only in Germany); however, there is a lack of rigorous, randomised, controlled studies of this drug and other treatments such as verapamil and topiramate, which are used off-label for CH attack prevention [6, 10, 11].

The PREVention and Acute treatment of chronic cluster headache (PREVA) study of non-invasive vagus nerve stimulation (nVNS) (gammaCore^®^; electroCore, LLC; Basking Ridge, NJ, USA) used adjunctively with standard of care (SoC) is the largest cCH prophylaxis trial to show significant treatment effects [6, 12]. The primary end point of PREVA was achieved, demonstrating a significantly more pronounced reduction from baseline in weekly attack frequency with nVNS + SoC than with SoC alone and yielding a mean therapeutic benefit of 3.9 fewer attacks per week (*P* = 0.02) [12]. Secondary end points were also met for patients in the nVNS + SoC group; 40% had a ≥50% reduction in weekly attack frequency, and a 57% reduction from baseline in abortive medication/oxygen use was observed (*P* < 0.001) [12]. Economic analyses of PREVA from German and UK perspectives demonstrated that nVNS + SoC was more cost-effective when compared with SoC alone [13].

An increasing interest in clinically informative and patient-centric outcomes, including the time to and level of therapeutic response, has been identified in the literature [4, 14, 15]. These outcomes are improving the ability of health care professionals and payers to assess the clinical significance of observed treatment benefits [4, 14, 15]. Randomised controlled studies of several emerging therapies for primary headache have expanded the definition of *responder rate* to include various levels of response (i.e. ≥25%, ≥50%, ≥75%, and 100%) [14–17]. A treatment’s capacity for faster onset and higher levels of response may be of particular importance for patients with CH because of the excruciating nature and therapeutic urgency of the associated pain [18, 19]. Here, we report a post hoc analysis of PREVA to further investigate the time to therapeutic benefit onset and the response rate levels associated with adjunctive nVNS used in cCH prophylaxis. This analysis allows clinicians to justify continued treatment for responders identified within a defined period.

## Methods

### Study design

A complete description of the methods for the 3-phase, multicentre, prospective, randomised, controlled PREVA study has been reported elsewhere [12]. After a 2-week baseline phase, in which all participants received their individualised SoC therapy, patients were randomly assigned (1:1) to receive nVNS + SoC or SoC alone during a 4-week randomised phase. An optional 4-week extension phase followed, with all patients receiving nVNS + SoC.

### Study population

Patients were 18 to 70 years of age and had been diagnosed with cCH according to *International Classification of Headache Disorders (ICHD)* criteria [4] more than 1 year before enrolment. Patients who had a change in prophylactic medication type or dosage less than 1 month before enrolment were excluded, as were those with a history of intracranial/carotid aneurysm, haemorrhage, surgery (e.g. carotid endarterectomy or vascular neck surgery), syncope, or seizures. Other key exclusion criteria were significant head trauma, known or suspected cardiac/cardiovascular disease, and current implantation with electrical or neurostimulation devices or metallic hardware.

### Intervention

Throughout PREVA, no changes in a patient’s prophylactic regimen were allowed. The nVNS-treated patients self-administered three 2-minute prophylactic stimulations (each separated by a period of 5 min) to the right side of the neck (right vagus nerve); this preventive treatment regimen occurred twice daily for a total of 6 stimulations per day. Three additional nVNS stimulations were permitted as needed for the acute treatment of individual CH attacks. Patients were permitted to receive abortive medications if their CH attacks persisted beyond 15 min after stimulation.

### End points

Mean weekly attack frequency over time, global percentage change from baseline in weekly CH attack frequency at the end of the randomised phase, and response rates in the randomised phase were evaluated in this post hoc analysis. Cut-offs of ≥25%, ≥50%, ≥75%, and 100% reductions from baseline in attack frequency were used to define *response*. The ≥50% response rate was a secondary end point of the PREVA study [12]. The remaining response rates were defined specifically for this post hoc analysis.

### Statistical analyses

All end points in this post hoc analysis were evaluated using a *modified intent-to-treat (mITT*) *population*, defined as subjects who had available data for each study week. For mean weekly attack frequency and global percentage change in weekly attack frequency, *P* values were derived from the *t* test. For response rates, *P* values were derived from the Fisher exact test or the chi-square test as appropriate.

## Findings

### Patients

**Fig. 1:**
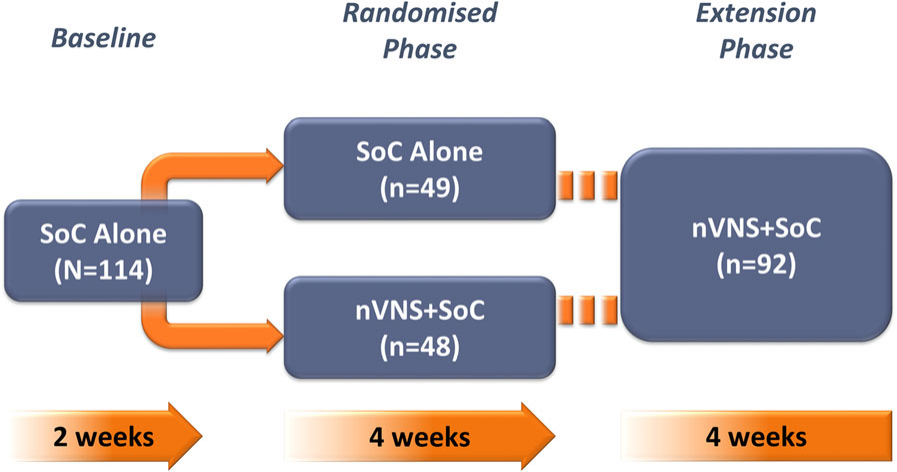
PREVA Study Design. Abbreviations: nVNS, non-invasive vagus nerve stimulation; SoC, standard of care

Complete descriptions of patient disposition, demographics, and baseline characteristics in PREVA have been reported previously [12]. A total of 97 patients with cCH were randomly assigned to receive nVNS + SoC (*n* = 48) or SoC alone (*n* = 49) (Fig. 1). Demographics, baseline characteristics, and use of prophylactic SoC medications (i.e. verapamil, lithium, topiramate, and corticosteroids) were comparable between groups (Table 1). The number of patients in the mITT population varied among the end points because of its dependence on the availability of measurable observations. Of the 92 patients who continued into the extension phase, 44 continued to receive nVNS + SoC and 48 switched from SoC alone to nVNS + SoC.

**Table 1:** PREVA Demographics and Baseline Characteristics

Characteristic	nVNS + SoC (*n* = 48)	SoC Alone (*n* = 49)
Age (y), mean ± SD	45.4 ± 11.0	42.3 ± 11.0
Sex (male), No. (%)	34 (71)	33 (67)
Time since cCH onset (y), mean ± SD	4.7 ± 3.9	5.0 ± 3.7^a^
CH attack duration (min), mean ± SD		
With acute pharmacologic medications/oxygen	27.4 ± 19.8	29.3 ± 29.9^b^
Without acute pharmacologic medications/oxygen	95.2 ± 57.7^c^	103.3 ± 66.8
Number of CH attacks in the 4 weeks before enrolment, mean ± SD	67.3 ± 43.6^c^	73.9 ± 115.8
Use of prophylactic CH medications, No. (%)		
Verapamil/verapamil hydrochloride	25 (52)	26 (53)
Lithium/lithium carbonate	6 (13)	9 (18)
Topiramate	7 (15)	7 (14)
Corticosteroids	2 (4)	2 (4)
Use of acute CH medications/oxygen, No. (%)		
Pharmacologic medications	43 (90)	44 (90)
Oxygen	32 (67)	34 (69)

*Abbreviations*: *CH* cluster headache, *nVNS* non-invasive vagus nerve stimulation, *SD* standard deviation, *SoC* standard of care

^a^Data were missing for 2 patients in the SoC alone group. ^b^Data were missing for 1 patient in the SoC alone group. ^c^Data were missing for 1 patient in the nVNS + SoC group. Adapted with permission from: Gaul C, Diener HC, Silver N, et al. (2016) Non-invasive vagus nerve stimulation for PREVention and Acute treatment of chronic cluster headache (PREVA): a randomised controlled study. Cephalalgia 36:534–546. Copyright 2016 SAGE Publishing

### Weekly attack frequency

**Fig. 2:**
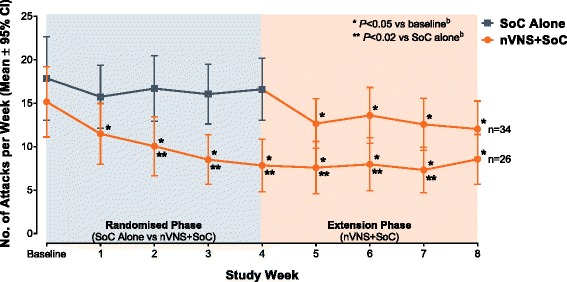
Mean Attack Frequencies (mITT Population^a^). Abbreviations: CI, confidence interval; mITT, modified intent-to-treat; nVNS, non-invasive vagus nerve stimulation; SoC, standard of care. ^a^ Subjects with available data for each study week. ^b^ From the *t* test

The mean weekly attack frequency was significantly lower with nVNS + SoC than with SoC alone from week 2 of the randomised phase through week 3 of the extension phase (*P* < 0.02; Fig. 2). For the nVNS + SoC group, attack frequencies were significantly reduced from baseline beginning at week 1 of the randomised phase and continuing through week 4 of the extension phase (*P* < 0.05). Attack frequencies were relatively stable throughout the extension phase.

**Fig. 3:**
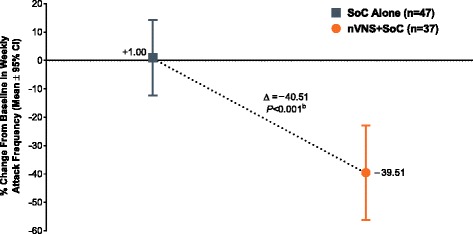
Global Change in Weekly Attack Frequency at the End of the Randomised Phase (mITT Population^a^). Abbreviations: CI, confidence interval; mITT, modified intent-to-treat; nVNS, non-invasive vagus nerve stimulation; SoC, standard of care. ^a^ Subjects with available data for each study week. ^b^ From the *t* test

Global mean attack frequency at the end of the randomised phase had decreased by 40% from baseline in the nVNS + SoC group and had increased by 1% with SoC alone, representing a 41% therapeutic benefit of nVNS (*P* < 0.001; Fig. 3).

### Response rates

**Fig. 4:**
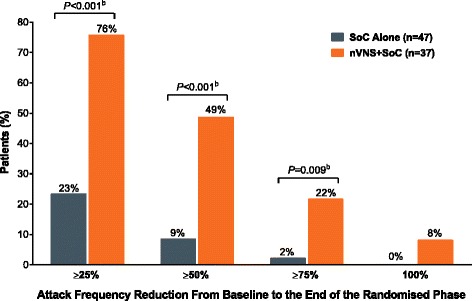
Response Rates (mITT Population^a^). Abbreviations: mITT, modified intent-to-treat; nVNS, non-invasive vagus nerve stimulation; SoC, standard of care. ^a^ Subjects with available data for each study week. ^b^ From the Fisher exact or chi-square test as appropriate

At the end of the randomised phase, a significantly higher percentage of patients in the nVNS + SoC group than in the SoC group had ≥25%, ≥50%, and ≥75% attack frequency reductions from baseline (≥25% and ≥50%, *P* < 0.001; ≥75%, *P* = 0.009; Fig. 4). There were 3 patients (8%) in the nVNS + SoC group who had a 100% attack frequency reduction; no patients who received SoC alone had a 100% response.

### Safety and tolerability

As previously reported [12], nVNS was safe and well tolerated in the PREVA study. There were similar proportions of patients in the nVNS + SoC and SoC groups who reported ≥1 adverse event. Rates of discontinuation due to adverse events were also similar between groups. No serious device-related adverse events occurred.

## Discussion

Our post hoc analysis of the PREVA study helps to further define the possible clinical value of the nVNS efficacy observed in the largest study of cCH prophylaxis to date that showed significant treatment effects [6, 12]. Significant beneficial effects were seen with nVNS + SoC (versus SoC alone) within 2 weeks after nVNS initiation. The SoC control group began to experience significant treatment benefits within 1 week after switching to nVNS + SoC. The potential for further benefits with continued nVNS treatment is consistent with findings from several studies [12, 20–23]. Significant reductions from baseline in weekly attack frequency were observed by the end of the first week of treatment and were sustained until the end of the study (i.e. week 8). In the 4-week randomised phase, nVNS + SoC treatment led to a 40% reduction in CH attack frequency, whereas SoC alone resulted in a 1% increase. The ≥25%, ≥50%, and ≥75% response rates were significantly higher with nVNS + SoC than with SoC alone. Limitations of this analysis are similar to those that have been well documented for post hoc analyses in general [24].

As important as the statistically significant findings, nVNS had effects that were *clinically meaningful*, defined as the ability to provide practical advantages that address current therapeutic challenges [25]. The excruciating nature of CH attacks warrants a greater sense of urgency for prophylactic treatment [18, 19] that may be addressed by nVNS with its rapid onset of efficacy, which was significant within 2 weeks of nVNS addition to SoC, and its beneficial effects on ≥25%, ≥50%, ≥75%, and 100% response rates. For the nVNS + SoC group, the time to response is as fast as that seen in a previous clinical study of patients with episodic CH treated with verapamil, which is also considered to have an early onset of effect once therapeutic levels are reached [18, 26]. Nearly half of patients treated with nVNS during the randomised phase of PREVA experienced a ≥50% response, which exceeds the 30% improvement that is widely accepted by general pain specialists as clinically meaningful [27–29]. The ≥25% response experienced by the majority of patients (76%) in the current analysis would likely also be considered clinically meaningful for those suffering from the intense pain of cCH.

Our analysis has identified subgroups of patients with dramatic responses similar to those seen in studies of other therapies for primary headache [14, 16, 17]. In the nVNS + SoC group, 8% of patients were attack free for the duration of the randomised phase, and no patients receiving SoC alone had this result. A ≥75% reduction in weekly attacks occurred in 22% of patients receiving nVNS + SoC compared with 2% of patients receiving SoC alone. Further efforts towards identifying potential predictors of such favourable responses may help to individualise future treatment decisions regarding adjunctive nVNS prophylaxis.

The safety, tolerability, and practicality of nVNS are well established [12, 22, 23, 30–33]. Based on the frequent pharmacologic dosing and potentially indefinite treatment period required for cCH, important challenges of prophylaxis include the potential for drug interactions and drug-related adverse events including atrioventricular conduction abnormalities, tremor, and confusion [18]. In PREVA, nVNS was easily incorporated into the existing pharmacologic SoC regimens without any risk of drug interactions or drug-related adverse events [12]. The safety and tolerability profile of nVNS may also help to avoid potential delays in pain relief, as seen in certain patients treated with verapamil, which requires gradual titration in order to minimize the risk of third-degree atrioventricular block [18, 34]. The significant reduction in abortive pharmacologic medication/oxygen use in the nVNS + SoC group of PREVA helps to mitigate the drug-related safety and tolerability, dosing, and portability challenges of existing acute CH therapies [7, 12, 35, 36]. Although PREVA did not examine the effects of nVNS in patients with episodic CH, the rapid beneficial effects on attack frequency observed within 2 weeks of treatment initiation in this cCH analysis, combined with the established safety profile of nVNS, suggest that a trial in episodic CH would be clinically reasonable.

## Conclusions

In this post hoc analysis of attack frequency over time and expanded response rates, the practical combination of nVNS and currently available cCH treatments led to rapid, sustained, and clinically meaningful responses. Within 2 weeks after the addition of prophylactic nVNS to SoC, sustained reductions in attack frequency were significantly greater with this combination than with SoC alone. After 4 weeks, patients’ average weekly attack frequency was 41% lower with prophylactic nVNS + SoC treatment than with SoC alone. The rapid decrease in weekly attack frequency justifies a 4-week trial period to identify responders to nVNS, with a high degree of confidence, among patients with cCH. The ≥25%, ≥50%, and ≥75% response rates were significantly higher with adjunctive nVNS than with SoC alone.

### Preva study group

Investigators are listed by country. 1. Germany: Migraine and Headache Clinic, Königstein – Charly Gaul, MD, PhD (principal investigator), and Ronald Brand, MD (subinvestigator); University Hospital-Essen, Essen – Hans-Christoph Diener, MD, PhD (principal investigator), and Kasja Rabe, Holle Dagny, Steffen Nägel, MD, and Maja Bak, MD (subinvestigators); Ludwig-Maximilian University, Munich – Andreas Straube, MD (principal investigator), and Bernhard Blum, MD, Ruth Ruscheweyh, MD, and Ozan Eren, MD (subinvestigators); Department of Neurology, Charité University Hospital, Berlin – Uwe Reuter, MD (principal investigator), and Heike Israel-Willner, MD, and Lars Neeb, MD (subinvestigators); Krankenhaus Lindenbrunn, Lindenbrunn – Stefan Evers, MD, PhD (principal investigator); 2. United Kingdom: The Walton Centre for Neurology and Neurosurgery, Liverpool – Nicholas Silver, MBBS, PhD (principal investigator), and Helen Banks, MD, and Heike Arndt, MD (subinvestigators); The Southern General Hospital, Glasgow – Alok Tyagi, MD (principal investigator); Hull Royal Infirmary, Hull – Fayyaz Ahmed, MD (principal investigator), and Anwar Osman, MD (subinvestigator); 3. Belgium: Liège University, Liège – Delphine Magis, MD, PhD (principal investigator), and Jean Schoenen, MD (subinvestigator); 4. Italy: Sant’Andrea Hospital, Sapienza University of Rome, Rome – Paolo Martelleti, MD (principal investigator), and Andrea Negro, MD (subinvestigator).

## Abbreviations

cCH: Chronic cluster headache
CH: Cluster headache
CI: Confidence interval
mITT: Modified intent-to-treat
nVNS: Non-invasive vagus nerve stimulation
PREVA: The PREVention and Acute treatment of chronic cluster headache study
SD: Standard deviation
SoC: Standard of care
